# 

**Published:** 1957-04

**Authors:** 


### Contents

| | | Page |
|---|---|---|
| SOME NOTES OF A MEDICAL VISITOR TO THE BRITISH WEST INDIES | *Professor A. V. Neale* | 33 |
| FROM BLUE TO PINK | *Dr. E. G. Bradbeer* | 46 |
| THE PORT HEALTH SERVICE | *Dr. D. T. Richards* | 56 |
| THE SYMPTOMS AND TREATMENT OF ACUTE FOLLICULAR TONSILLITIS | *Dr. S. G. Flavell Matts* | 63 |
| ASTROCYTOMA TREATED BY RADIOTHERAPY: DEATH FROM ACUTE LEUKAEMIA | *Clinical Pathological Conference* | 67 |
| NOTES AND NEWS | | 73 |
| APPOINTMENTS | | 73 |

